# Association of Serum Bilirubin and Functional Variants of Heme Oxygenase 1 and Bilirubin UDP-Glucuronosyl Transferase Genes in Czech Adult Patients with Non-Alcoholic Fatty Liver Disease

**DOI:** 10.3390/antiox10122000

**Published:** 2021-12-15

**Authors:** Jaromír Petrtýl, Karel Dvořák, Jan Stříteský, Martin Leníček, Alena Jirásková, Václav Šmíd, Martin Haluzík, Radan Brůha, Libor Vítek

**Affiliations:** 1 4th Department of Internal Medicine, 1st Faculty of Medicine, Charles University and General University Hospital in Prague, 120 00 Prague, Czech Republic; petrtyl@hotmail.com (J.P.); karel.dvorak@nemlib.cz (K.D.); venca.smid@gmail.com (V.Š.); 2 Institute of Pathology, 1st Faculty of Medicine, Charles University and General University Hospital in Prague, 120 00 Prague, Czech Republic; Jan.Stritesky@vfn.cz; 3 Institute of Medical Biochemistry and Laboratory Diagnostics, 1st Faculty of Medicine, Charles University and General University Hospital in Prague, 120 00 Prague, Czech Republic; mleni@centrum.cz (M.L.); a.jiraskova@email.cz (A.J.); halm@ikem.cz (M.H.); 4 Institute of Clinical and Experimental Medicine, 140 00 Prague, Czech Republic

**Keywords:** NAFLD, NASH, bilirubin, bilirubin UDP-glucuronosyl transferase, *UGT1A1*, heme oxygenase 1, *HMOX1*, oxidative stress

## Abstract

Non-alcoholic fatty liver disease (NAFLD) is the most prevalent chronic liver disorder worldwide. The aim of our study was to assess the role of bilirubin, and the heme oxygenase 1 (*HMOX1*) and bilirubin UDP-glucuronosyl transferase (*UGT1A1*) promoter gene variants, which are involved in bilirubin homeostasis, in the NAFLD development in adult patients. The study was performed on 84 patients with NAFLD and 103 age/sex-matched controls. Routine biochemistry, inflammatory markers, adipokines, and the fibrosis/steatohepatitis stage were determined in the NAFLD patients. The (GT)n/(TA)n dinucleotide variations in *HMOX1*/*UGT1A1* gene promoters, respectively, were analyzed by fragment analysis. Compared to controls, serum bilirubin concentrations in NAFLD patients tended to be decreased, while the prevalence of phenotypic Gilbert syndrome was significantly low. Genetic variations in *HMOX1* and *UGT1A1* gene promoters did not differ between NAFLD patients and controls, and no relationship was found in the NAFLD patients between these gene variants and any of the laboratory or histological parameters. In conclusion, metabolism of bilirubin is dysregulated in NAFLD patients, most likely due to increased oxidative stress, since frequencies of the major functional variants in the *HMOX1* or *UGT1A1* gene promoters did not have any effect on development of NAFLD in adult patients.

## 1. Introduction

Non-alcoholic fatty liver disease (NAFLD) constitutes the hepatic manifestation of the metabolic syndrome, affecting a large proportion of the population worldwide [1]. NAFLD is comprised of a spectrum of conditions from simple steatosis, non-alcoholic steatohepatitis (NASH), to liver cirrhosis—with a dramatic impact on population health [2].

While simple steatosis is usually considered a benign condition, NASH is already a potentially progressive disease of the liver, which might develop into liver fibrosis and ultimately to cirrhosis [3]. The factors leading to the progression of liver disease in selected patients with NAFLD are not completely understood. The initial model considered the development of simple steatosis as a “first hit”, increasing the sensitivity of the liver to hepatocyte injury (leading to inflammation and fibrosis) by a “second hit” [4]. The factors acting as a “second hit” were considered to be increased oxidative stress, elevated cytokines (like TNF-α), and reactive oxygen species (ROS)-mediated lipid peroxidation [5]. In fact, data from animal models and human studies show that mitochondria are the most important source of ROS in NASH [6], and oxidative cellular damage was demonstrated in the livers of patients with NASH [7]. The degree of oxidative stress is determined by disequilibrium between prooxidant and antioxidant factors. Unlike the factors leading to oxidative stress that are generally known in patients with NASH, the protective factors are not well recognized, and specific genetic predispositions might play an important role in the modulation of this balance [8]. The enzyme with a potential protective effect against oxidative damage of liver cells is heme oxygenase 1 (*HMOX1*, OMIM*141250), which is responsible for bilirubin production. The *HMOX1* induction in an experimental animal model of NASH led to suppression of inflammation and fibrosis, as well as improvement in hepatic morphology. The *HMOX1* gene promoter contains a highly polymorphic (GT)n sequence, the longer (GT)n repetition (classified as the L allele) being associated with decreased HMOX activity and increased risk of oxidative stress-mediated diseases [9]. The role of functional variants of the *HMOX1* gene for NAFLD evolution is not clear, although a recent Taiwanese study suggested that the presence of the L allele might be a risk factor for NAFLD, at least in the Asian population [10]. This is consistent with the observation of increased *HMOX1* expression in the liver tissue of NASH patients, believed to be an adaptive response against oxidative damage accompanying NASH [11]. These data are supported by experimental observations in which a higher expression of *HMOX1* has been associated with less severe NAFLD disease progression in animal models [12,13].

Another important enzyme with a potentially protective effect is bilirubin UDP-glucuronosyl transferase (*UGT1A1*, OMIM*191740), responsible for bilirubin biotransformation, whose gene promoter (containing (TA)n dinucleotide repeats) is also highly polymorphic. Subjects with variants responsible for the manifestation of Gilbert syndrome are protected from the development of diseases associated with increased oxidative stress, provided that they are accompanied with elevated bilirubin levels [14]. However, in a substantial proportion of individuals this protection is not present, since the penetrance of Gilbert syndrome genotype is only 50% [15]. Nevertheless, in one study from Taiwan, the protective effects of serum bilirubin concentrations and *UGT1A1* variants were described in pediatric patients with NAFLD with an independent negative association between variant UGT1A1*6 genotypes (prevalent in Asian populations) and NAFLD prevalence [16]. The possible predictive role of *UGT1A1* variants in the development of NAFLD in adult patients, and in other populations, so far has not been reported.

It is generally acknowledged that the major biological effector of *HMOX1* and *UGT1A1* gene variants is bilirubin, which is a potent molecule with versatile metabolic functions in the human body [17]. In fact, serum bilirubin concentrations have been reported to be negatively associated with NAFLD/NASH in adults [18,19,20] as well as in pediatric patients [21]. On the other hand, two recent Mendelian randomization studies from China [22] and the Netherlands [23] did not prove any causal association of systemic bilirubin concentrations and NAFLD; and in the recent Italian study, no association of serum bilirubin with liver steatosis was observed either [24]. Moreover, in another study by Tarantino et al., serum bilirubin concentrations were higher across all stages of NAFLD [25], and NASH patients were shown to have higher serum bilirubin concentrations compared to those with simple steatosis [26].

Hence, the aim of the present study was to assess the role of bilirubin, and the *HMOX1* and *UGT1A1* promoter gene variants, which are involved in bilirubin homeostasis, in the NAFLD development in adult patients.

## 2. Materials and Methods

### 2.1. Patients

The study was performed on a group of 84 patients with NAFLD. These were consecutive patients examined between 2010 and 2013 at the 4th Department of Internal Medicine, General University Hospital in Prague. The diagnosis of NAFLD was based on liver histology in 56 individuals, or on clinical and laboratory parameters according to AASLD practice guidelines: (1) evidence of hepatic steatosis, either by imaging or liver biopsy [27]; and (2) lack of secondary causes of hepatic fat accumulation [2]. Exclusion diagnoses included the following: viral hepatitis, drug-induced liver disease, biliary diseases, autoimmune liver disease, and inherited metabolic disorders, as well as acute infection illnesses. Alcohol abuse was excluded by the patients’ history, a short questionnaire, and a laboratory examination (stable, non-fluctuating GGT activities, and examination of serum carbohydrate deficient transferrin (CDT) or urine ethyl-glucuronide in subjects with suspected alcohol abuse). The diagnosis of NASH was based on liver histology (see below). The control group consisted of 103 age- and sex-matched healthy individuals (employees or blood donors from the Transfusion Unit of the of the General University Hospital in Prague) without liver and/or coronary artery disease, or other chronic diseases.

The study was carried out in accordance with the Helsinki Declaration, and approved by the Ethics Committee of the General University Hospital in Prague.

### 2.2. DNA Analysis

Genomic DNA was isolated from peripheral blood white cells by a standard salting-out method. The (GT)n variations in the *HMOX1* (dbSNP rs1805173), and (TA)n variations in the *UGT1A1* (dbSNP rs81753472) gene promoters were simultaneously determined using multicolored capillary electrophoresis as previously described [28]. Corresponding DNA fragments were amplified by polymerase chain reaction (PCR) using the following primers: (for *HMOX1*: forward 5′-CTGCAGCTTCTCAGATTTCC-3′; reverse 5′-ACAAAGTCTGGCCATAGGAC; for *UGT1A1*: forward 5′-GAACTTGGTGTATCGATTGGTTTTGC-3′; reverse 5′-CATCCACTGGGATCAACAGTATCTTCC-3′). The reverse primers were labeled at the 5′ end by WellRED fluorescent dyes (Beckman Coulter, Fullerton, CA, USA). PCR products were separated on a CEQ 8000 Genetic Analysis System (Beckman Coulter, Fullerton, CA, USA). The length variations of *HMOX1* (GT)n repeats were classified into short S (*n* < 27), medium M (*n* = 27–32), and long L (*n* = 33) subgroups.

### 2.3. Liver Biopsy

A liver biopsy was available in 56 patients, conducted in 43 patients by the percutaneous method with a Menghini needle, and by the transjugular method in 13 patients. The indication for transjugular biopsy was obesity, thrombocytopenia, or a suspicion of liver cirrhosis and the need for a hepatic venous pressure gradient measurement. The biopsy samples were routinely stained and read by a single pathologist (JS), who was blind to the clinical and laboratory data. NAFLD/NASH was evaluated according to the NAFLD activity score (NAS): Steatosis (0–2), lobular inflammation (0–2), hepatocellular ballooning (0–2), and fibrosis (0–4) [29].

### 2.4. Laboratory Methods

All routine serum biochemical markers were determined on an automatic analyzer (Modular analyzer, Roche Diagnostics GmbH, Mannheim, Germany) using standard laboratory assays. CDT and urine ethyl glucuronide were determined by high-performance liquid chromatography. Inflammatory cytokines (IL-2, IL-6, and TNF-α) were analyzed by Luminex technology using a multiplex kit (Linco Res, St. Charles, MO, USA). Adiponectin, leptin, and insulin were measured by ELISA (Roche Diagnostics, Indianapolis, IN, USA). Serum hyaluronic acid (HA) was measured by a latex agglutination method (HA LT, Latex Agglutination Method, Wako Chemicals GmbH, Neuss, Germany). M30 and M65 cytokeratin-18 fragments were determined by commercially available ELISA tests (PEVIVA AB, Sweden). Non-invasive liver fibrosis scoring systems were calculated according to available formulae: APRI [30], FIB-4 [31], NAFLD fibrosis score [32], and BARD score [33].

### 2.5. Statistical Methods

The data are expressed as the mean ± SD, or median (IQ range) when the data were non-normally distributed. T-Test or Mann-Whitney Rank Sum Test were used to compare the laboratory parameters of patients with NAFLD and controls. Allele frequency was evaluated by the Chi-square test. The Kruskal-Wallis test and ANOVA tests were used to compare clinical and laboratory parameters in different genotypes for *HMOX1* and *UGT1A1* gene promoters. All analyses were performed with alpha set to 0.05. The statistics were computed using STATISTICA CZ v. 12 (StatSoft, Prague, Czech Republic) and BMDP Statistical Software (Release 8.1).

## 3. Results

### 3.1. Serum Bilirubin Concentrations and Liver Enzyme Activities in NAFLD Patients and Control Population

The basic characteristics of the studied groups are demonstrated in Table 1. In a subgroup of patients who underwent liver biopsy, NASH was diagnosed in 38 patients (68% of patients), while simple steatosis was found in 18 patients (32%). Patients with NASH suffered from more advanced fibrosis compared to those with simple steatosis (mean fibrosis stage 2.64 ± 0.9 vs. 1.3 ± 1.4; *p* = 0.002).

As expected, NAFLD patients had increased BMI, fasting glucose, and insulin concentrations, as well as elevated ALT and GGT activities; all markers of metabolic syndrome and NAFLD (Table 1). In contrast, a trend toward lower serum concentrations of bilirubin was observed in NASH patients (predominantly in men), although this difference did not reach statistical significance (Table 1 and Table 2). Interestingly, NASH patients had a much lower prevalence of phenotypic Gilbert syndrome (defined as hyperbilirubinemia > 17 μmol/L, with liver enzyme activity within the physiological range) when compared with the control population: 1.9% vs. 16.9% in males, and 3.8% vs. 15.6% in females, with *p* < 0.05 for both comparisons.

### 3.2. Analysis of Functional Variants of HMOX1 and UGT1A1 in Patients with NAFLD and Control Population

Since both *HMOX1* and *UGT1A1* are involved in the defense against increased oxidative stress, we had aimed to assess whether the genetic variations associated with either enhanced *HMOX1* activity (responsible for increased bilirubin production), or decreased *UGT1A1* activity (responsible for sluggish bilirubin biotransformation), are less prevalent in NAFLD patients.

Quite surprisingly, no significant difference in the frequencies of analyzed genotypes of *HMOX1* and *UGT1A1* genes was found between the patients with NAFLD and the control population (Table 3). Similarly, no difference was found in the frequency of the above-mentioned gene variations when they were analyzed separately by gender (data not shown).

Presence of the *HMOX1* L allele did not affect serum bilirubin concentrations, but as expected *UGT1A1* (TA)_7_ gene variation significantly increased serum bilirubin concentrations in both NAFLD patients and controls (Table 2). Control subjects with (TA)_7/7_ gene variations (UGT1A1*28 allele homozygosity, typical for the manifestation of Gilbert syndrome in the Caucasian population) had higher serum bilirubin concentrations compared to the same homozygotes from the NAFLD group (Table 2).

We then analyzed the possible association between *HMOX1* and *UGT1A1* genotypes with laboratory markers of liver disease, inflammation, or fibrogenesis. No association of *HMOX1* and *UGT1A1* gene variants was found for any of the various laboratory markers tested (Tables S1 and S2), the stage of liver fibrosis, or the presence of NASH (data not shown), thus indicating a negligible role of these polymorphisms on the clinical status of NAFLD patients.

The inflammatory markers studied did not discriminate NAFLD patients according to their fibrosis status. The only exception was IL-6, whose concentration was significantly higher in F4 NAFLD patients compared to other fibrosis groups (Table 4).

## 4. Discussion

Bilirubin, the major product of the heme catabolic pathway, is believed to contribute importantly to the defense against increased oxidative stress [34]. Its systemic concentrations, which are negatively associated with numerous oxidative stress-mediated diseases [35], are mainly regulated by the highly polymorphic *UGT1A1* and *HMOX1* genes [36].

Nevertheless, our observations did not confirm the expectation that specific functional variations in *HMOX1* and *UGT1A1* gene promoters could have a protective effect in the development of NAFLD. Differences were neither found in the inflammatory parameters among patients with different *HMOX1/UGT1A1* variants, nor in the severity of liver disease.

Despite previous findings demonstrating the beneficial effects of increased expression of *HMOX1* on NAFLD development, and progression in experimental settings as well as a human study on Taiwanese pediatric NAFLD patients, our observations in adult patients with a substantial proportion of advanced liver disease did not confirm these data.

In addition, we were not able to find any association between the Gilbert UGT1A1*28 genotype and NAFLD. Similarly, no such association was found in the pediatric study from Taiwan (where, however, the UGT1A1*28 gene variant is not the major mutation responsible for Gilbert syndrome manifestation) [16]. On the other hand, the Taiwanese authors found an association of pediatric NAFLD and the UGT1A1*6 gene variant, which is prevalent in Asian populations [16]. It should also be stressed that the penetrance of the UGT1A1*28 gene mutation is only 50% [15], which certainly confounds results from the gene association studies. Our negative results are also in concordance with another of our studies on patients with chronic HCV infection, in which no association was found between variations of *UGT1A1* and/or *HMOX1* genes and HCV infection-induced liver injury [37]. Although *UGT1A1* is the major gene determining serum bilirubin concentrations [36], it was clearly shown that in obese children the percentage of body fat is a more important predictor of total serum bilirubin concentration, independent of the UGT1A1*28 polymorphism [38]. This fact is certainly highly relevant to all bilirubin studies on patients with NAFLD.

It has been reported repeatedly that individuals with Gilbert syndrome and increased bilirubin levels have a lower frequency of cardiovascular diseases, cancer, as well as various metabolic diseases compared to normobilirubinemic subjects [35,39]. However, as described above, the epidemiological data on the association of serum bilirubin and NAFLD/NASH reported so far are controversial [18,19,20,21,22,23,24,25,26]. The results from our clinical study did not find any such association, as was similarly found in recently published studies [22,23,24,25,26]. The major confounding factor might be NAFLD-induced liver dysfunction, which might even lead to a certain elevation of serum bilirubin concentration. However, in this setting, the possible protective effect of bilirubin is being lost [40]. Thus, the most likely explanation for almost equal serum bilirubin concentrations in NASH patients and control subjects is the latent-to-apparent liver damage (as evidenced by increased ALT activities (Table 1)), and the dysregulated oxidative stress defense (as evidenced by increased GGT activities (Table 1)) of the liver parenchyma in NASH patients that is accompanied with impaired hepatic bilirubin metabolism and a mild elevation of serum bilirubin concentrations (Table 1 and Table 2). In fact, a substantial proportion of the NAFLD patients examined in our study suffered from various degrees of liver fibrosis (regardless of the fact that the serum bilirubin concentrations did not correlate with hepatic fibrosis stage, Table 4), thus corroborating the above-mentioned explanation.

In addition, bilirubin was demonstrated to correlate well with fibrosis in NAFLD patients, with increasing bilirubin concentrations being associated with a worse stage of NAFLD [41]. Lower, as well as higher, bilirubin concentrations (most likely as a reflection of liver damage due to underlying NAFLD; also see above) were reported to be associated with the presence of NASH in another pediatric study [42]. According to that study, higher serum bilirubin was found to be a positive predictive factor in a novel NashTest, introduced by the French authors [43]; and its positive role for predicting liver fibrosis in NAFLD patients was also confirmed by German scientists in their Non-Invasive Koeln-Essen Index (NIKEI) [44]. Interestingly, the same predictive role of elevated serum bilirubin concentrations has also been found in other studies [32,45,46]. Although bilirubin has not been included in the NAFLD fibrosis score, it was demonstrated to be an independent predictor of fibrosis in a univariate analysis [32], and a similar positive role of bilirubin for predicting liver fibrosis in patients with NAFLD was also demonstrated in both FibroTest and Actitest liver fibrosis scoring systems [45], as well as in the similar clinical study on NAFLD patients by Stepanova et al. [46].

These facts are in accord with our observation of the dramatically low prevalence of a phenotypic Gilbert syndrome (defined as unconjugated hyperbilirubinemia with no elevation of liver enzyme activities) in our NAFLD patients, suggesting that those with elevated liver enzymes have almost entirely increased serum bilirubin concentrations due to liver function deterioration in advanced NAFLD.

## 5. Conclusions

In conclusion, our data demonstrate a dysregulated metabolism of bilirubin in adult NAFLD patients, most likely due to increased oxidative stress accompanying NAFLD, since the frequencies of the major functional variants in the *HMOX1* or *UGT1A1* gene promoters did not demonstrate to have any major effect on development of NAFLD.

## Figures and Tables

**Table 1:** Clinical and laboratory parameters of patients with NAFLD and controls.

Parameter	NAFLD (*n* = 84)	Controls (*n* = 103)	*p*-Value
**Age** (years)	50.7 ± 14.2	43.9 ± 9	ns
**Males/Females** (No.)	59/25	62/41	ns
**BMI** (kg/m^2^)	30.7 ± 3.6	26.6 ± 3.5	<0.001
**Fasting glucose** (mmol/L)	5.9 ± 1.4	4.7 ± 0.4	<0.001
**Bilirubin** (μmol/L)	10.5 (7.8–16.7)	12.5 (9–15.4)	ns
**ALT** (μkat/L)	0.94 (0.5–1.8)	0.43 (0.3–0.5)	<0.001
**GGT** (μkat/L)	1.25 (0.7–2.4)	0.38 (0.3–0.6)	<0.001

Depending on the data distribution, data are expressed as mean ± SD or median and interquartile range. Comparisons were performed using a *t*-test or Mann–Whitney Rank Sum test, respectively.

**Table 2:** Serum bilirubin in patients with NASH and controls, also subdivided according to *HMOX1* and *UGT1A1* promoter genotypes.

	Serum Bilirubin (μmol/L)		
	Cases	Controls	*p-*Value ^a^
**all**	10.5 (7.8–16.7)	12.5 (9–15.4)	ns
**males**	10 (7.9–16.9)	13 (9.4–15.9)	ns
**females**	11 (7.7–16)	10 (8.7–13.3)	ns
***HMOX1*** **(GT)n status**			
**L allele**	15 (9.7–17.4)	12.3 (9.1–21.4)	ns
**S/M allele**	10.5 (7.5–16.8)	12.6 (9–15.3)	ns
***p*** **-Value**	ns	ns	
***UGT1A1*** **(TA)n status**			
**(TA)_6/6_**	8.3 (6.2–11)	10.9 (8.4–13.1)	ns
**(TA)_6/7_**	13.1 (8.3–16.8)	12.5 (9.1–15.3)	ns
**(TA)_7/7_**	17.7 (8.2–18.8)	19.9 (15–25.6)	0.05
***p*** **for trend**	0.02	<0.001	

Data are expressed as median and interquartile range. Comparisons were performed using a Mann–Whitney Rank Sum test, while comparisons within cases and controls for *HMOX1* (GT)n status were performed using analysis of variance (ANOVA) on ranks with Dunn’s *post hoc* analysis. ^a^ Differences in serum bilirubin among individual genotypes in cases and controls. *UGT1A1*, bilirubin UDP-glucuronosyl transferase; ns, not significant.

**Table 3:** Frequency of functional variants of *HMOX1* and *UGT1A1* in patients with NAFLD and control population.

Genotype	NAFLD (*n* = 84)	Controls (*n* = 103)	*p*-Value
***HMOX1***			
**M/M**	29 (35%)	29 (28%)	
**M/L**	6 (7%)	7 (6.5%)	
**S/L**	2 (2%)	6 (5.5%)	ns
**S/M**	37 (44%)	50 (49%)	
**S/S**	10 (12%)	10 (10%)	
**L/L**	0	1 (1%)	
***UGT1A1 ^#^***			
**5/6 + 6/6**	26 (32%)	37 (36%)	ns
**5/7 + 6/7**	45 (55%)	51 (50%)	
**7/7**	11 (13%)	15 (14%)	
**6/7 + 7/7**	55 (67%)	65 (63%)	ns *

Data expressed as number of patients (%). *p* value represents the difference in frequencies of all genotypes between patients and control subjects using a Chi-square test. * = vs. 5/6 + 6/6 genotype. *^#^* DNA for *UGT1A1* examination available only from 82 patients. The length variations of *HMOX1* (GT)n repeats were classified into short S (*n* < 27), medium M (*n* = 27–32), and long L (*n* = 33) subgroups. The distribution of the studied gene variations was in Hardy–Weinberg equilibrium.

**Table 4:** Inflammatory parameters and adipokines in patients with different stages of liver fibrosis.

Parameter	F0 (*n* = 16)	F1 (*n* = 13)	F2 (*n* = 10)	F3 (*n* = 9)	F4 (*n* = 8)	*p*-Value
**Bilirubin** (µmol/L)	9.7 (7.3–16.9)	14.2 (8–18.2)	8.6 (7.5–16.8)	9.4 (8.2–10.6)	13 (8.7–25.1)	ns
**IL-2** (ng/L)	0.72 ± 1	0.93 ± 0.7	17.5 ± 51	4.13 ± 9.1	16.9 ± 29	ns
**IL-6** (ng/L)	1.5 (0.7–4.2)	3.1 (2.2–7.8)	4.3 (1.9–19.9)	1.1 (0.9–1.3)	15.2 (5.7–76)	<0.05 *
**TNF-α** (ng/L)	6.44 ± 3.7	10.3 ± 4.4	10.2 ± 6.3	7.2 ± 5	15.7 ± 10.9	ns
**Adiponectin** (mg/L)	5.3 (4.7–8.4)	4.7 (2.9–8.8)	3.6 (3.2–6.6)	8.8 (2–15.7)	7 (3.5–28.1)	ns
**Leptin** (μg/L)	10.9 (8.6–15.4)	7.8 (4.5–10.5)	11.6 (5.6–18.3)	13.6 (2–25.2)	5.8 (4.8–22.5)	ns

Data are expressed as mean ± SD or median and interquartile range depending on data distribution. Depending on data normality, comparisons were performed using analysis of variance (ANOVA) or ANOVA on ranks with Dunn’s post hoc analysis. * F4 vs. F0–F3.

## Data Availability

All research data are available on request from the Corresponding Author. We do not have webpages created for such purpose, but as we state the data are available upon request and can be sent to anyone interested in our research.

## Supplementary Materials

The following are available online at www.mdpi.com/article/10.3390/antiox10122000/s1, Table S1: Laboratory and clinical parameters in patients with different *HMOX1* promoter genotypes. Table S2: Laboratory and clinical parameters of patients with different *UGT1A1* promoter genotypes.

## Abbreviations

*HMOX1*	heme oxygenase 1
NAFLD	non-alcoholic fatty liver disease
NASH	non-alcoholic steatohepatitis
*UGT1A1*	bilirubin UDP-glucuronosyl transferase 1A1
