# Supplementary material for: Association of Serum Bilirubin and Functional Variants of Heme Oxygenase 1 and Bilirubin UDP-Glucuronosyl Transferase Genes in Czech Adult Patients with Non-Alcoholic Fatty Liver Disease
Source: Antioxidants (Basel). 2021 Dec 15;10(12):2000. doi: 10.3390/antiox10122000 (PMC8698489; doi:10.3390/antiox10122000)
Supplement: Supplementary file 1 [file antioxidants-10-02000-s001.zip › antioxidants-1479193-supplementary.pdf]

Table S1. Laboratory and clinical parameters in patients with different *HMOX1* promoter genotypes.

Parameter	*HMOX1* genotype				*P*-value
	M/L (n=6)	M/M (n=29)	S/M (n=37)	S/S (n=10)	
**Bilirubin** (µmol/l)	15 (9.9-17.9)	13.1 (7.6-18.2)	9.1 (6.9-14.7)	13.2 (9.1-14.7)	ns
**GGT** (µkat/l)	4.39 (0.93-5.5)	1.66 (0.63-2.97)	1.24 (0.98-2.05)	0.99 (0.62-1.57)	ns
**ALT** (µkat/l)	1.25 (0.63-2.07)	0.69 (0.46-2.2)	0.96 (0.65-1.8)	0.5 (0.39-1.72)	ns
**AST** (µkat/l)	0.87 (0.5-1.24)	0.62 (0.45-1.08)	0.67 (0.52-1.07)	0.45 (0.39-0.93)	ns
**Albumin** (g/l)	47.2 (45.1-48.4)	45.3 (37.3-46.4)	47.3 (44.4-49.1)	46.2 (44.6-47.6)	ns
**BMI** (kg/m^2)	29.4 (28.4-29.9)	30.6 (27.7-33.7)	29.6 (28.3-31.6)	32.5 (27.8-37.5)	ns
**IL-2** (ng/l)	2.55 (0.14-18.9)	0.54 (0.14-1.2)	0.61 (0.3-4.2)	0.34 (0.1-0.9)	ns
**IL-6** (ng/l)	16.44 (4.9-25.9)	4.83 (2-15.2)	4.27 (1.9-18)	5.18 (2.2-29.1)	ns
**TNF-α** (ng/l)	6.22 (5.6-6.9)	9.5 (7.3-12.2)	9.1 (6.3-13.4)	8.19 (6.3-17.2)	ns
**Adiponectin** (mg/l)	5.0 (3.9-9.8)	5.2 (3.5-12.3)	4.5 (2.7-7.5)	8.8 (4.3-14.1)	ns
**Leptin** (µg/l)	8.3 (4.6-13.4)	10.2 (5.8-18.7)	7.4 (4.5-11.6)	8.6 (5.5-18.4)	ns
**HA** (µg/l)	33.4 (18.6-70.3)	22.5 (11.9-38.9)	24 (16.6-34.3)	19.2 (16-137)	ns
**M30** (U/l)	7.29 (6.2-404)	72.2 (9.9-292)	13 (8.5-176)	184.5 (13.4-292)	ns
**M65** (U/l)	534 (375-1085)	833 (417-1310)	572 (407-1126)	647 (441-900)	ns
**AST/ALT**	0.72 (0.57-0.9)	0.7 (0.56-1.13)	0.61 (0.56-0.9)	0.76 (0.5-0.95)	ns
**APRI**	0.52 (0.3-0.8)	0.55 (0.3-0.8)	0.5 (0.3-1)	0.5 (0.2-0.8)	ns
**FIB 4**	1.07 (1-1.8)	1.51 (1-1.9)	1.04 (0.8-1.9)	1.22 (0.7-2.2)	ns
**NAFLD fibrosis score**	-2.1 (-4.3--1.4)	-1.56 (-2.2-(-0.3))	-2.43 (-3.1-(-0.2))	-2.71 (-3.9-(-0.15))	ns
**BARD score**	1 (0-1.75)	2 (1-3)	1 (1-3)	1 (0-3)	ns

Data are expressed as the median and IQ range. Analyzed by ANOVA; ns, not significant.

The length variations of *HMOX1* (GT)n repeats were classified into short S (n < 27), medium M (n = 27–32), and long L (n = 33) subgroups. S/L genotype was not assessed due to the small number of patients (n=2).

Table S2. Laboratory and clinical parameters of patients with different *UGT1A1* promoter genotypes.

Parameter	*UGT 1A1* genotype			*P* for trend
	**6/6** (n=26)	**6/7** (n=45)	**7/7** (n=11)	
**Bilirubin** (μmol/l)	8.3 (6.2-11)	13.1 (8.3-16.8)	17.7 (8.2-18.8)	0.02
**GGT** (μkat/l)	1.85 (0.7-3.4)	1.3 (0.7-2.7)	1.21 (0.8-1.8)	ns
**ALT** (μkat/l)	0.98 (0.5-1.8)	0.85 (0.5-2.3)	0.94 (0.5-1.4)	ns
**AST** (μkat/l)	0.62 (0.5-1)	0.67 (0.5-1.2)	0.55 (0.4-0.8)	ns
**Albumin** (g/l)	45.6 (41-48)	45.9 (43-48)	47.6 (46-49)	ns
**BMI** (kg/m^2)	29.6 (27.6-31.3)	30.1 (28.9-34.9)	31.2 (29.6-33.6)	ns
**IL-2** (ng/l)	0.23 (0.13-0.83)	0.87 (0.21-2.6)	0.47 (0.21-6.1)	ns
**IL-6** (ng/l)	4.05 (1.1-11.3)	5.88 (2.2-21.3)	7.83 (2.2-17.7)	ns
**TNF-α** (ng/l)	8.52 (6.3-11.9)	9.06 (6.3-12.8)	8.76 (4.8-16.4)	ns
**Adiponectin** (mg/l)	4.8 (3.5-7.1)	6.3 (3.3-11.5)	3.7 (3-5.2)	ns
**Leptin** (μg/l)	7.1 (2.6-9.9)	8.8 (6.5-18.7)	9.3 (4.2-15.9)	ns
**HA** (μg/l)	23.7 (18-35)	24.9 (11-46)	19.9 (17-26)	ns
**M30** (U/l)	23.4 (9-297)	14.8 (8-267)	12.7 (8-336)	ns
**M65** (U/l)	542 (453-1026)	784 (375-1363)	602 (378-1393)	ns
**AST/ALT**	0.6 (0.6-1)	0.77 (0.6-0.9)	0.66 (0.6-0.9)	ns
**APRI**	0.5 (0.3-0.9)	0.57 (0.3-0.9)	0.37 (0.3-0.6)	ns
**FIB 4**	1.36 (0.8-2.2)	1.32 (0.9-1.9)	0.97 (0.8-1.2)	ns
**NAFLD fibrosis score**	-2.08 (-4.2-(-0.03))	-2.18 (-3.2-(-0.9))	-1.61 (-2.3-(-0.6))	ns
**BARD score**	1 (0-3)	2 (1-3)	1 (1-2.25)	ns

Data are expressed as the median and IQ range. Comparisons were performed using analysis of variance (ANOVA) on ranks with Dunn's *post hoc* analysis. ns, not significant

6/7 genotype group also covers one subject with 5/7 genotype
